# Associations Between Masseter Muscles Tension, Postural Pattern, Pelvic Type and Postural Stability in Women with Pelvic Organ Prolapse

**DOI:** 10.3390/jcm15218283

**Published:** 2026-10-24

**Authors:** Małgorzata Wójcik, Kamil Góral, Jolanta Małysz, Magdalena Kubryń, Artur Hołodniuk, Danuta Lietz-Kijak, Idzi Siatkowski, Grażyna Jarząbek-Bielecka, Piotr Skomro, Bartosz Aniśko

**Affiliations:** 1 Department of Physiotherapy, Faculty of Physical Culture in Gorzow Wielkopolski, Poznan University of Physical Education, 61-871 Poznan, Poland; k.goral@awf-gorzow.edu.pl (K.G.); mkubryn96@wp.pl (M.K.); a.holodniuk@awf-gorzow.edu (A.H.); b.anisko@awf-gorzow.edu.pl (B.A.); 2 Conocimiento Student Research Association, Faculty of Sport Sciences in Gorzow Wielkopolski, Poznan University of Physical Education, 61-871 Poznan, Poland; jolantamalysz@gmail.com; 3 Department of Propaedeutic, Physical Diagnostics and Dental Physiotherapy, Faculty of Medicine and Dentistry, Pomeranian Medical University, 70-204 Szczecin, Poland; danuta.lietzkijak@gmail.com (D.L.-K.);; 4 Statistical Methods, Poznan University of Life Science, 60-637 Poznan, Poland; idzi.siatkowski@up.poznan.pl; 5 Division of Gynecology, Department of Gynaecology, Poznan University of Medical Sciences, 61-758 Poznan, Poland; grajarz@o2.pl

**Keywords:** pelvic organ prolapse, postural stability, masseter muscle, women

## Abstract

**Background**: This study aimed to examine the associations of pelvic type and body posture pattern with masseter muscle measurements and postural stability parameters in women with POP, and to assess the relationship between masseter muscle measurements and postural stability under eyes-open and eyes-closed conditions. **Methods**: This observational study included 61 women: 31 with advanced POP (grade 3–4 according to the Pelvic Organ Prolapse Quantification system [POP-Q]) and 30 healthy controls. Masseter muscle measurements, postural stability parameters (center-of-pressure sway and surface area), body posture pattern, and pelvic type were assessed. Postural stability was measured with eyes open and closed. Two-way analysis of variance (ANOVA), Tukey’s post-hoc test, and Pearson’s correlation coefficient were used for statistical analysis. Results: Pelvic type was not significantly associated with any of the analyzed parameters. Body posture pattern was associated with significant differences in most postural stability parameters (*p* < 0.05) under both visual conditions, with the highest values observed in women with a posterior posture pattern. **Conclusions**: In women with advanced POP, body posture pattern was associated with differences in postural stability parameters, whereas no statistically significant associations were observed for pelvic type. No statistically significant correlations were detected between masseter muscle measurements and postural stability. These findings should be interpreted cautiously, given the observational study design, and do not establish causal relationships.

## 1. Introduction

Physiotherapeutic urogynecological diagnostics require a detailed medical anamnesis and a holistic approach, taking into account the relationships between internal organs, the musculoskeletal system, and postural stability [1]. The biological and clinical mechanisms underlying the relationship between pelvic organ prolapse (POP), body posture, and postural stability are not yet fully understood. This relationship may be explained by myofascial chains and the mechanisms of postural control. Referring to the myofascial chains described by Myers, there is fascial continuity in the body that provides a connection between the masseter muscles, the pelvic floor muscles, and the feet [2]. In vivo studies provide partial support for epimuscular myofascial force transmission (EMFT). In vitro findings suggest that fascial tissue may transmit up to 30% of mechanical loads; however, evidence in humans remains limited. Fascia is considered a structure that coordinates movement and body stability [3].

Pelvic organ prolapse is increasingly understood not merely as an isolated anatomical defect, but as a consequence of impaired coordination between pelvic floor muscles and the broader trunk stabilization system [3]. It has been shown that the pelvic floor muscles increase their tone prior to limb movement [4].

The concept proposed by Lewit suggests that pathological processes within internal organs may manifest as a viscerosomatic reflex (increased muscle tone, trophic changes, and fascial alterations in skin temperature and moisture) in the musculoskeletal system [4], which in turn justifies extending diagnostic assessment to include the craniomandibular system in women with pelvic organ prolapse.

The masseter muscles have a direct influence on the function of the temporomandibular joints, playing a key role in occlusion, chewing, and mandibular movements [5]. The temporomandibular joints and pelvic floor muscles are functionally connected through the deep front line (myofascial chain). Excessive tension or dysfunction within the pelvic floor muscles may be associated with increased tension in the temporomandibular joints and vice versa [6]. As demonstrated, a single soft tissue therapy session in the temporomandibular joint region can improve pelvic floor muscle function [6]. There is also a relationship between body posture and temporomandibular joint disorders [7].

The pelvis in the human body constitutes the foundation of the postural system, and its alignment determines the biomechanics of the entire body, influencing posture and muscle activation through fascial chains and neuromuscular control [8]. Pelvic alignment affects the timing of Anticipatory Postural Adjustments (APA), the activation of the transversus abdominis and multifidus muscles, as well as global trunk coordination [4].

Recent research suggests that the pelvic floor muscles participate not only in urogynecological functions, but also in postural control and the regulation of intra-abdominal pressure. However, it remains poorly understood whether, in women with advanced pelvic organ prolapse, global posture pattern, pelvic type, and parameters of the stomatognathic system are associated with postural stability. The literature regarding the relationship between the stomatognathic system and body posture remains inconclusive, and most available studies concern dental or orthodontic populations, or individuals with temporomandibular joint disorders, rather than women with POP.

Pelvic organ prolapse (POP) is a condition in which one or more pelvic organs descend from their normal position because the muscles and ligaments of the pelvic floor have become weakened or damaged. It is also referred to as pelvic floor prolapse or pelvic organ descent. Therefore, there is a lack of studies simultaneously assessing masseter muscle tension, posture pattern, pelvic type, and static postural stability in women with advanced POP, particularly under both eyes-open and eyes-closed conditions. Postural stability was assessed with both eyes open and eyes closed, as visual input provides information about the body’s position relative to the surrounding environment.

Postural patterns described by Hall, Wernham, and Littlejohn have been reported to influence the relationship with the stomatognathic system, including the tension of the masticatory muscles [9,10]. Three postural patterns are distinguished: neutral (normal), anterior, and posterior [9,10].

The anterior postural pattern is characterized by increased cervical lordosis, elevated tension in the posterior spinal muscles and ligaments, increased loading of the lumbosacral junction, increased diaphragmatic tension, and excessive stretching of the abdominal muscles. This pattern may contribute to a predisposition for visceral ptosis and irritation within the pelvic region [9,10]. In contrast, the posterior postural pattern is characterized by increased loading of the cervicothoracic junction, increased thoracic kyphosis, increased lumbar lordosis, and greater stress on the sacroiliac joints. It is also associated with increased diaphragmatic tension, elevated intra-abdominal pressure affecting the abdominal and pelvic organs, and a predisposition to constipation [9,10].

According to the classification proposed by Gutmann and Erdmann, different pelvic types are characterized by distinct sacral inclination and pelvic alignment, which influence lumbar curvature and the distribution of mechanical loads [4,9]. Although no direct evidence has demonstrated an association between these pelvic types and postural stability, altered pelvic orientation may affect lumbopelvic load transfer, trunk muscle activity, and postural control through biomechanical mechanisms.

The aim of our study was to determine whether, in women with pelvic organ prolapse, there is a relationship between masseter muscle tension and postural stability with respect to posture patterns described by Hall and pelvic types described by Gutmann and Erdmann.

Before commencing the study, the following research hypotheses were formulated:

**H1.**  *Masseter muscle tension is associated with postural stability parameters measured under both eyes-open and eyes-closed conditions in women with pelvic organ prolapse.*

**H2.**  *Masseter muscle tension differs between individuals with different pelvic types and postural patterns, as reflected by postural stability parameters.*

**H3.**  *The relationships between masseter muscle tension, pelvic type, postural pattern, and postural stability parameters may differ between eyes-open and eyes-closed conditions.*

## 2. Methods

### 2.1. Study Design

The observational study was conducted as a single assessment. Women with a diagnosis of pelvic organ prolapse (POP) were included in the study group if they achieved a score of 3–4 according to the Pelvic Organ Prolapse Quantification scale (POP-Q) [11], along with healthy women with no diagnosed gynecological conditions, including pelvic organ prolapse (POP), in the control group. All measurements were performed by an experienced physiotherapist. The study was conducted after obtaining approval from the Bioethics Committee of Poznań University of Medical Sciences (No. 305/23) on 11 May 2023 and was registered in ClinicalTrials.gov under the identifier NCT05978414 on 7 July 2023.

This study employed a cross-sectional observational design to investigate the associations between masseter muscle tension, pelvic type, postural pattern, and postural stability under eyes-open and eyes-closed conditions. Registration with ClinicalTrials.gov took place before the first participant was enrolled, and the methodology and the results analysed are in accordance with the registered protocol.

### 2.2. Participants

The study included 61 women, including 31 with a medical diagnosis of POP, with a mean age of 50 ± 7.07 years (Table 1). Women with POP were assessed by a gynecological specialist using the POP-Q scale, obtaining a stage 3–4 classification. The study also included 30 healthy women with a mean age of 47 ± 1.41 years (Table 1), who served as the control group.

The inclusion criteria for the study were: pelvic organ prolapse with a POP-Q stage of 3–4, and written informed consent to participate in the study in accordance with the Declaration of Helsinki.

The exclusion criteria were: the presence of clicking or popping and/or pain in the temporomandibular joint(s), ongoing dental treatment, use of dental prostheses/bridges, abdominal hernias, recent abdominal surgery, arterial hypertension, current physiotherapy treatment, cancer, and lack of written informed consent to participate in the study.

Before measurements were conducted, all participants were informed about the procedure. Women with pelvic organ prolapse (POP) underwent an initial assessment using the POP-Q classification system. Participants classified as stage 3 or 4 according to the POP-Q system were eligible for inclusion in the subsequent phase of the study.

All women in both the pelvic organ prolapse group and the control group underwent assessment of masseter muscle tension, postural stability (anterior–posterior sway, medio-lateral sway, and surface area) under both eyes-open and eyes-closed conditions. In accordance with Hall’s classification, body posture pattern was assessed, and pelvic type was evaluated according to Gutmann and Erdmann. The assessment was conducted by an experienced physiotherapist with 29 years of clinical practice.

All assessments were performed between 10:00 a.m. and 1:00 p.m. Participants were recruited during their clinical visits and invited to take part in the study.

### 2.3. Masseter Muscle Tension

The authors used a measurement device designed to assess masseter muscle tension. The device was developed by the authors (PAT 2568 W.132167; patent W.132167—consent of the Polish Patent Office ). Its construction consists of two parallel beams between which strain gauges are placed to record their deformation under applied compressive force. The signal from the strain gauges was transmitted to a force meter, which displayed the result in mass units (kilograms) [12]. The recorded result was entered into an Excel spreadsheet. Measurements were performed under both eyes-open and eyes-closed conditions. Each measurement lasted 30 s. A 5-min rest interval was applied between the eyes-open and eyes-closed measurements. The assessment was conducted in a standing position, with the upper limbs alongside the trunk, feet positioned at hip-width apart, and the participant looking straight ahead. During the measurement, the participant was instructed to contract the masseter muscles with maximal possible force. Each woman in both the POP and control groups performed the test once. The device was placed between the upper and lower teeth, and it was held by the examiner during the procedure. Before each measurement, the device was checked to ensure that the initial reading was 0.00 kg. The measurement took 30 s, as this was the duration of the postural stability assessment on the platform. To minimize measurement error, the device was tested on healthy individuals (the measurements were performed repeatedly) before measurements were taken in the female study participants. Before each measurement, the device was tared in both the test group and the group of women participating in the study by setting the display to 0.00 kg. The device used to assess masseter muscle function was developed by the authors and calibrated before data collection. However, independent criterion validation and test –retest reliability were not established. Furthermore, only one measurement was obtained from each participant, preventing the assessment of within-subject repeatability and measurement error. Therefore, the findings related to masseter muscle measurements should be interpreted cautiously, and further studies are needed to establish the device’s measurement accuracy, reliability, and validity.

Independent validation of the device could not be performed due to the lack of comparable reference devices, which represents a limitation of the present study. The results obtained suggest that the device may be useful for assessing muscle tension within the mandibular system. However, further studies are needed to confirm the repeatability of the measurements obtained. The group assignment of each participant remained concealed from the physiotherapist responsible for conducting the assessment. The measurements were performed by an experienced physiotherapist who was unaware that masseter muscle tension measurements would be analyzed in relation to postural type, pelvic pattern, and postural stability parameters.

### 2.4. Postural Stability Assessment

Postural stability parameters, i.e., anterior–posterior sway, medio-lateral sway, and the area of center of pressure (COP) displacement, were assessed using the Computerised Stability Platform CQStab2P-vUSB-1506 manufactured by the Polish company Koordynacja. The platform was subjected to a validation process to assess its accuracy, reliability, and the conformity of the results obtained with the established reference criteria. The measurement duration, rest interval, and testing conditions for eyes-open and eyes-closed trials were conducted in an analogous manner to the assessment of masseter muscles tension. During the study, the women stood barefoot on a platform (both feet were positioned parallel to each other and hip-width apart), with their upper limbs held alongside their torsos. The results obtained for the anterior-posterior and lateral-medial deviations were given in millimetres (mm). The surface area is given in the results in square millimetres (mm^2^). The obtained results were recorded in an Excel spreadsheet. The examiner was not informed of the participants’ group allocation, remaining unaware of whether they were assigned to the POP group or the control group. The measurements were performed by an experienced physiotherapist who was unaware that postural stability parameters would be analyzed in relation to postural type, pelvic pattern, masseter muscle tension, and postural stability parameters.

### 2.5. Hall’s Posture Pattern Assessment

The posture pattern according to Hall, Wernham, and Littlejohn is understood as a coherent whole. The interrelationships between the pelvis, the spine (lumbar, thoracic, and cervical regions), and the shoulder girdle are assessed [9]. During the assessment, the participant stood sideways in front of a corrective grid mirror, wearing only underwear and without footwear. An experienced physiotherapist evaluated posture by observing the alignment of the cervical, thoracic, and lumbar regions. After the assessment, the posture pattern was recorded in an Excel spreadsheet. A normal pattern is characterized by the axial alignment of the head, shoulders, and pelvis with preservation of spinal curvatures. In the anterior pattern, head and shoulder protraction and trunk flexion are observed. The posterior pattern is characterized by head and shoulder retraction and excessive trunk extension [9]. The assessing physiotherapist was also blind to whether the participants belonged to the POP group or the control group. The measurements were performed by an experienced physiotherapist who was unaware that postural pattern results would be analyzed in relation to pelvic type, masseter muscle tension, and postural stability parameters.

### 2.6. Gutmann and Erdmann’s Pelvic Type Assessment

Gutmann and Erdmann distinguished three pelvic types, indicating their influence on body statics [4]: a type with a high promontory and a long sacrum (the level of the iliac crests is above the level of the fifth lumbar vertebra, L5), in which the pelvis is positioned in posterior tilt; an intermediate type—neutral (the iliac crests are at the level of L5); and a load-related type with a low promontory and a large pelvic inclination (the iliac crests are below the level of L5), in which the pelvis is positioned in anterior tilt. The assessment of pelvic and spinal alignment (sacral inclination, pelvic position, and lumbar lordosis) is crucial for analyzing posture in the sagittal plane [13]. The examination can be performed in different positions; in our study, pelvic type was assessed in a standing position. The participant stood in underwear without footwear, and an experienced physiotherapist identified the L5 level by marking a point on the participant’s skin with a marker. The level of the iliac crests was then evaluated in relation to the L5 level. The resulting pelvic type classification was recorded in an Excel file. The assessing physiotherapist was also blind to whether the participants belonged to the POP group or the control group. The assessment of postural patterns or pelvic types is a commonly used examination performed by physiotherapists. The measurements were performed by an experienced physiotherapist who was unaware that pelvic type results would be analyzed in relation to postural pattern, masseter muscle tension, and postural stability parameters.

### 2.7. Data Analysis

For the statistical analysis, the normality of data distribution was first tested using the Shapiro–Wilk test. Since a normal distribution was confirmed, a two-way ANOVA test (without interaction, which proved to be statistically non-significant) was applied to examine whether pelvic type and posture pattern influenced masseter muscle tension and postural stability parameters (anterior–posterior sway, medio-lateral sway, and surface area) under both eyes-open and eyes-closed conditions, where the *p* value, statistical power, presumed sample size, and effect size (Eta-squared) were determined. To further determine which specific posture patterns and pelvic types respond similarly to masseter muscle tension and postural stability parameters, basic descriptive statistics were calculated. In addition, Tukey’s post hoc test was used for multiple comparisons. This test automatically adjusts the significance level (α) to account for multiple comparisons, thereby controlling the family-wise error rate (FWER). Thus, the probability of making at least one Type I error across the family of comparisons is maintained at the specified significance level. Pearson’s r correlation coefficient was applied to assess the strength of relationships between masseter muscle tension and postural stability parameters (anterior–posterior sway, medio-lateral sway, and surface area) under both eyes-open and eyes-closed conditions. All tests were two-sided, and *p* values ≤ 0.05 were considered significant. The results were presented in the form of tables and figures. The R statistical package version 4.5.2 was used for all analyses [14].

## 3. Results

Due to the small sample size, results that are not statistically significant should not be interpreted as conclusive evidence of a lack of association between the variables under study. In the group of women with pelvic organ prolapse, the following statistical analysis was performed:

### 3.1. Postural Stability—Anterior–Posterior Sway Eyes Open

Two-way ANOVA (Table 2 and Table 3) showed that pelvic type (pt) had no significant effect on anterior–posterior postural stability sway (*p* = 0.4927), whereas body posture pattern (bpp) had a significant effect (*p* = 0.0048), indicating its importance in sagittal-plane postural control. Tukey’s post-hoc analysis (Table 4) revealed significant differences between posterior and anterior posture patterns (*p* = 0.0053) and between posterior and neutral patterns (*p* = 0.0080), but not between neutral and anterior groups (*p* = 0.9591). Descriptive statistics (Table 5) showed the highest sway in the posterior posture group (mean = 442.0), indicating greater variability in postural stability compared with the other patterns.

### 3.2. Medio-Lateral Sway Eyes Open

As shown in Table 2, pelvic type was not significantly associated with medio-lateral postural stability sway (*p* = 0.3314), whereas body posture pattern showed a significant effect (*p* = 0.0244). According to the Tukey post-hoc analysis (Table 4), the posterior and anterior posture patterns differed significantly (*p* = 0.0215), while neither the neutral–anterior (*p* = 0.7658) nor posterior–neutral comparison (*p* = 0.0539) reached statistical significance. The descriptive data in Table 5 indicated the greatest medio-lateral sway for the posterior posture pattern (mean = 220.6667), suggesting higher variability in this group.

### 3.3. Surface Area Eyes Open

Table 2 shows that pelvic type (pt) was not significantly related to postural stability surface area (*p* = 0.5768), while body posture pattern (bpp) had a significant effect (*p* = 0.0132). Tukey’s post-hoc analysis (Table 4) identified significant differences between the posterior and anterior patterns (*p* = 0.0127) and between the posterior and neutral patterns (*p* = 0.0238), whereas the anterior–neutral comparison was not significant (*p* = 0.8931). As reported in Table 5, the posterior pattern had the highest mean surface area (1134.3333), indicating greater variability in postural stability.

### 3.4. Postural Stability—Anterior–Posterior Sway Eyes Closed

Two-way ANOVA (Table 2) showed no significant effect of pelvic type (pt) on postural stability anterior-posterior (*p* = 0.536), while body posture pattern (bpp) significantly affected this parameter (*p* = 0.016). Tukey’s post-hoc test (Table 4) showed significant differences between the posterior and anterior patterns (*p* = 0.0198) and between the posterior and neutral patterns (*p* = 0.0208), with no significant difference between the anterior and neutral patterns (*p* = 0.9993). Table 5 showed the highest mean anterior-posterior sway for the posterior pattern (699.6667), indicating greater postural stability variability in this group.

### 3.5. Medio-Lateral Sway Eyes Closed

Two-way ANOVA (Table 2) found no significant effect of pelvic type (pt) on medio-lateral sway (*p* = 0.6200), while body posture pattern (bpp) had a significant effect (*p* = 0.0004). Tukey’s test (Table 4) showed significant differences between the posterior pattern and both anterior (*p* = 0.0004) and neutral patterns (*p* = 0.0010), whereas the anterior–neutral comparison was not significant (*p* = 0.8622). According to Table 5, the posterior pattern had the highest mean sway (315), indicating greater postural stability variability in this group.

### 3.6. Surface Area (Eyes Closed)

The two-way ANOVA (Table 2) showed that pelvic type (pt) did not have a significant effect on center of pressure surface area (*p* = 0.3318), whereas body posture pattern (bpp) had a significant effect on this parameter (*p* = 0.0041), indicating its importance in shaping postural stability. The post-hoc Tukey test (Table 4) revealed significant differences between the posterior posture pattern and both the anterior (*p* = 0.0077) and neutral patterns (*p* = 0.0043). No significant differences were found between the neutral and anterior groups (*p* = 0.9167). Descriptive statistics (Table 5) showed the highest surface area values in the posterior posture pattern group, where the mean was 1742, indicating greater variability in postural stability in this group compared to the other posture patterns.

### 3.7. Masseter Muscles Tension Open Eyes

For the masseter muscles with eyes open, two-way ANOVA (Table 2) showed no significant effects of pelvic type (pt; *p* = 0.227) or body posture pattern (bpp; *p* = 0.279) on muscle tension. Tukey’s test (Table 4) also revealed no significant differences between posterior–anterior (*p* = 0.7460), posterior–neutral (*p* = 0.3860), or neutral–anterior (*p* = 0.5698) groups. Table 5 showed the highest mean tension in the neutral posture group (4.25), although the difference was not statistically significant.

### 3.8. Masseter Muscles Tension Eyes Closed

With eyes closed, two-way ANOVA (Table 2) showed no significant effect of pelvic type (pt; *p* = 0.521) or body posture pattern (bpp; *p* = 0.697) on masseter muscle tension. Tukey’s post-hoc analysis (Table 4) found no significant differences between posterior–anterior (*p* = 0.7220), posterior–neutral (*p* = 0.7066), or neutral–anterior (*p* = 0.9989) groups. As shown in Table 5, the neutral posture pattern had the highest mean tension (4.0071), although the difference was not statistically significant.

### 3.9. Correlation Analysis

The next stage of the statistical analysis involved determining the strength of the relationship between masseter muscle tension and postural stability parameters under both eyes-open and eyes-closed conditions using Pearson’s correlation coefficient (r). In addition, the statistical significance of the correlations was assessed.

**Figure 1:**
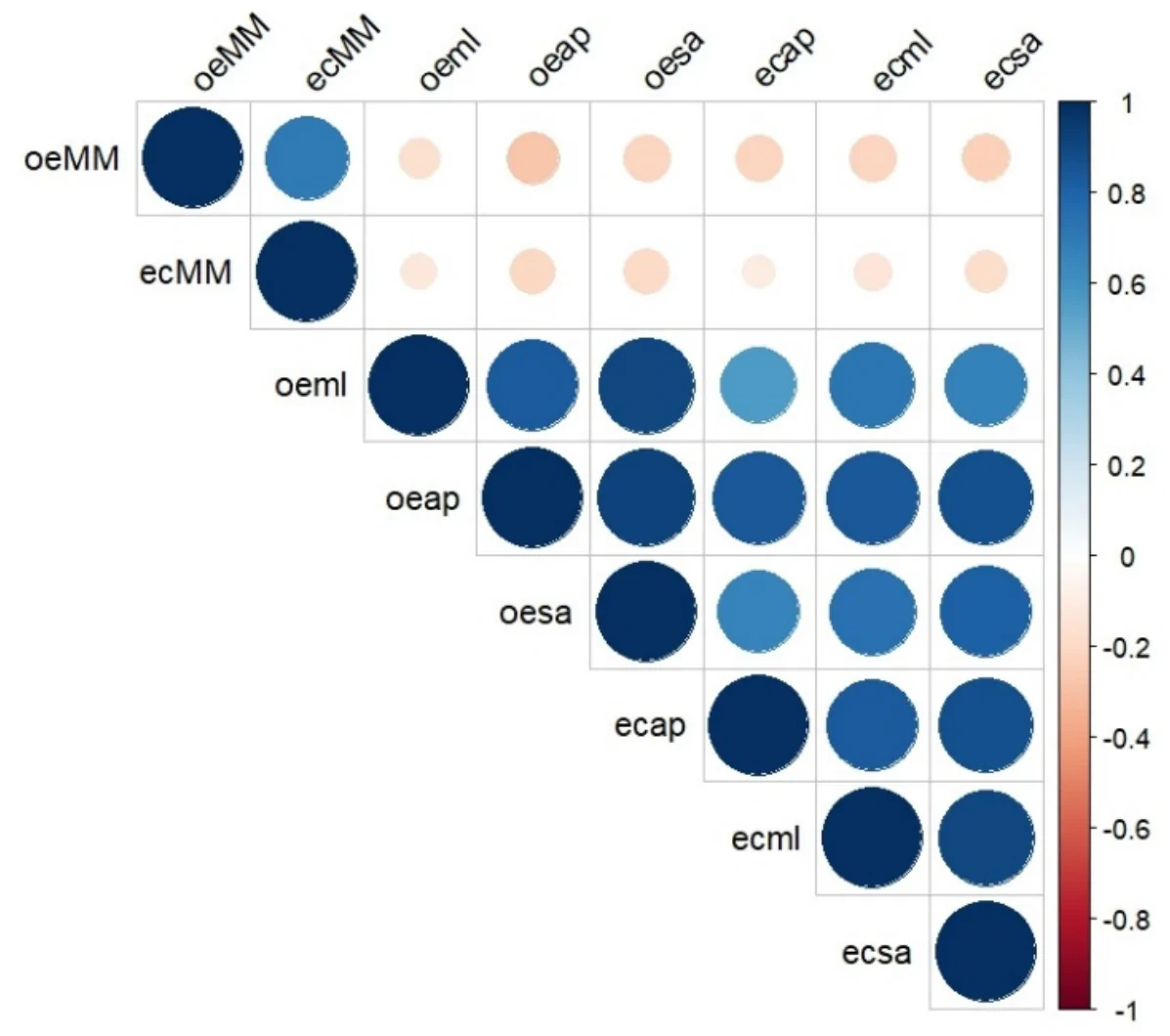
Pearson’s correlation coefficient (r) between masseter muscle tension and postural stability parameters under both eyes-open and eyes-closed conditions.

For the eyes-open condition, the following values of the correlation coefficient between masseter muscle tension and the analyzed parameters were obtained (Figure 1):

1. With anterior–posterior sway: r = −0.28 and *p*-value = 0.244, indicating a very weak, negative correlation and a statistically non-significant relationship between the analyzed parameters.
2. With medio-lateral sway: r = −0.17 and *p*-value = 0.363, also indicating a very weak, negative correlation and a statistically non-significant relationship between the analyzed parameters.
3. With surface area: r = −0.22 and *p*-value = 0.122, likewise indicating a very weak, negative correlation and a statistically non-significant relationship between the analyzed parameters.

For the eyes-closed condition, the following values of the correlation coefficient between masseter muscle tension and the analyzed parameters were obtained:

1. With anterior–posterior sway: r = −0.22 and *p*-value = 0.253, indicating a very weak, negative correlation and a statistically non-significant relationship between the analyzed parameters.
2. With medio-lateral sway: r = −0.22 and *p*-value = 0.496, also indicating a very weak, negative correlation and a statistically non-significant relationship between the analyzed parameters.
3. With surface area: r = −0.22 and *p*-value = 0.253, likewise indicating a very weak, negative correlation and a statistically non-significant relationship between the analyzed parameters.

Global observation of pelvic organ prolapse vs. control group

**Figure 2:**
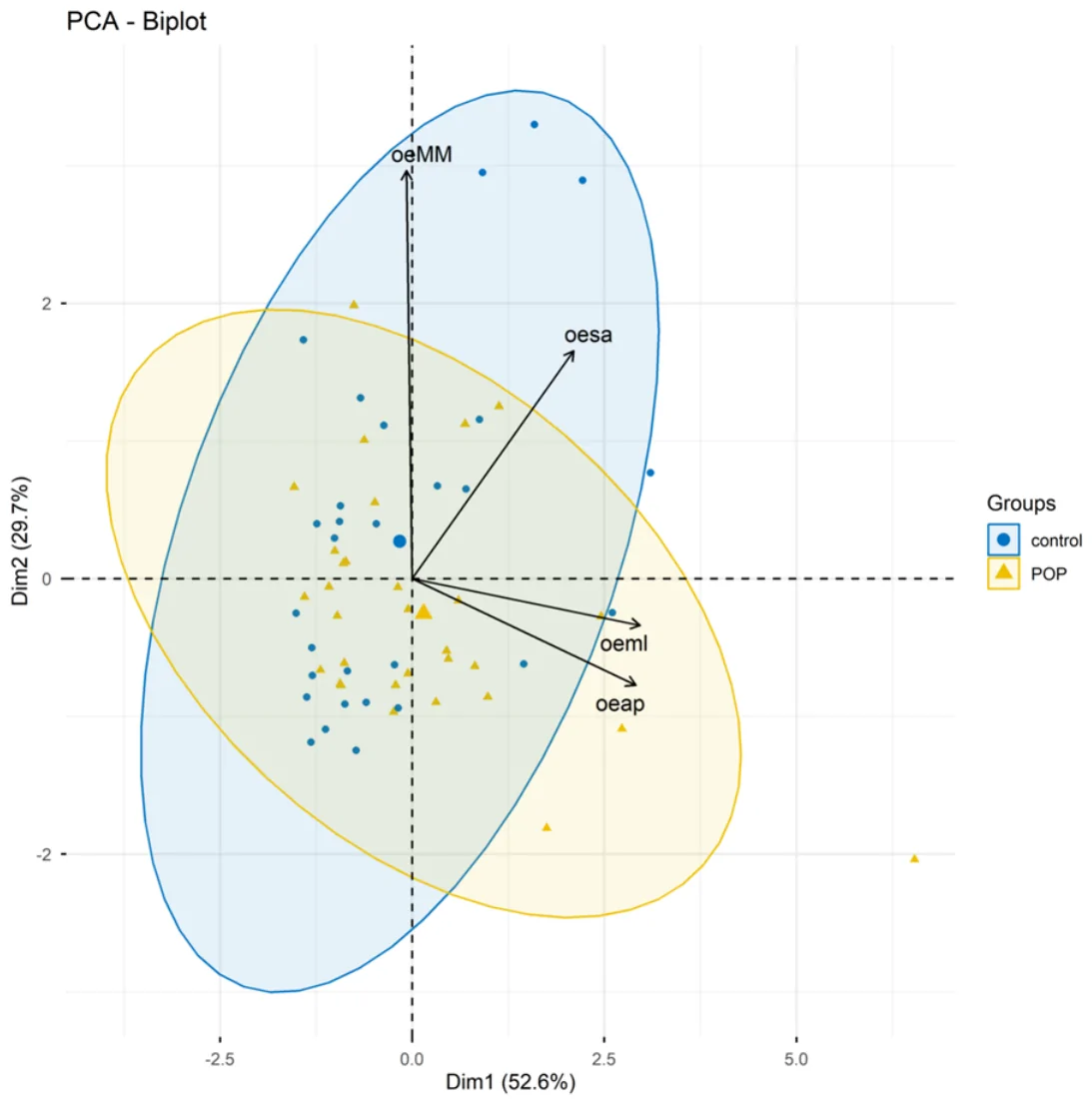
Simultaneous analysis of diaphragms in the pelvic organ prolapse group and the control group with eyes open.

**Figure 3:**
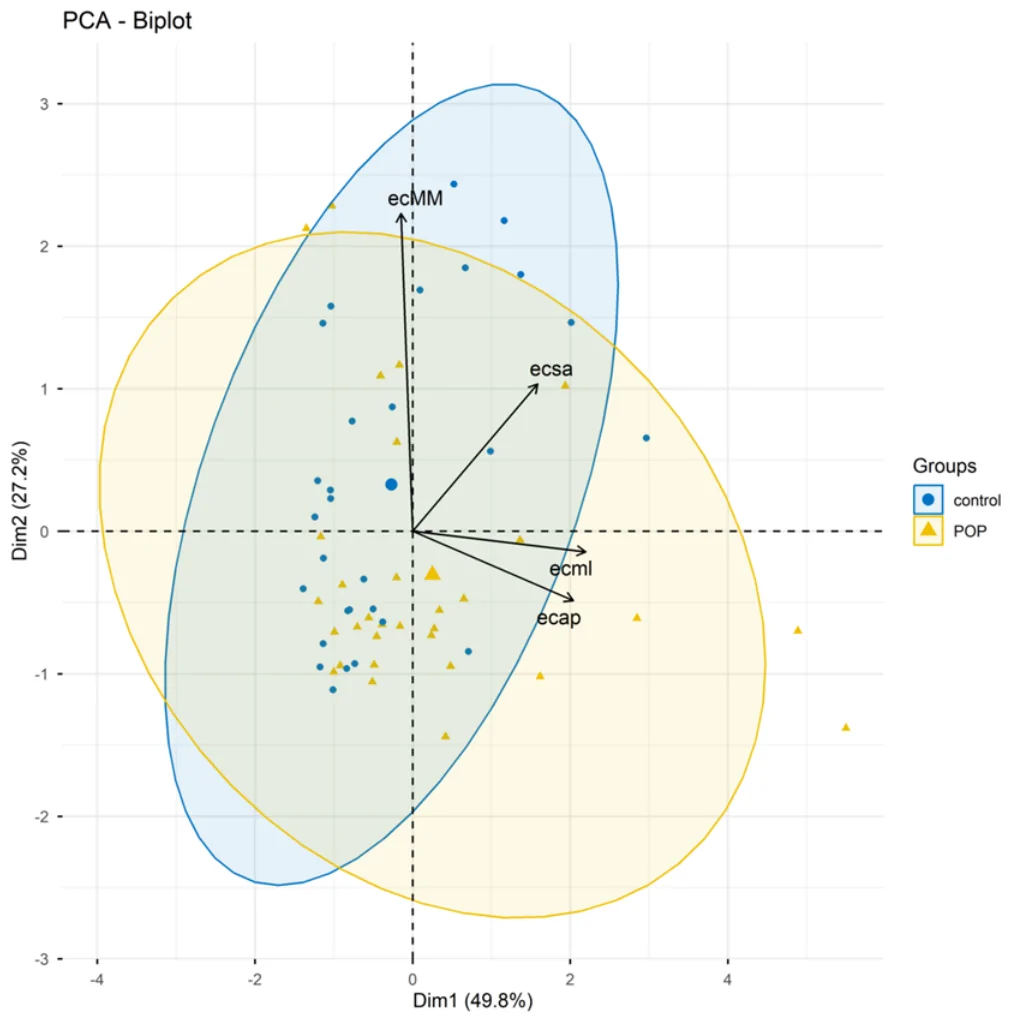
Simultaneous analysis of diaphragms in the pelvic organ prolapse group and the control group with eyes closed.

The final stage of the analysis involved a holistic approach to the problem by simultaneously analyzing all parameters for the examined functional diaphragms in the pelvic organ prolapse group and the control group under eyes-open conditions, and subsequently under eyes-closed conditions (Figure 2 and Figure 3).

The observational results of the diaphragms (circles and triangles) and their vectors indicate that the group of women with pelvic organ prolapse differs in terms of masseter muscle tension and postural stability parameters compared to the group of healthy controls, both under eyes-open and eyes-closed conditions (Figure 2 and Figure 3).

## 4. Discussion

Given the observational nature of the study, the obtained findings require a cautious and balanced interpretation regarding their clinical significance.

The aim of our study was to evaluate the relationship of pelvic type and body posture pattern on masseter muscle tension and postural stability parameters in women with pelvic organ prolapse, as well as to analyze the relationship between masseter muscle tension and postural control under both eyes-open and eyes-closed conditions.

The obtained results indicate that pelvic type was not significantly associated with postural stability parameters or masseter muscle tension. In contrast, body posture pattern showed significant differences in most stabilometric parameters.

This suggests that global postural organization may play a more important role in balance control than the pelvic types described by Gutmann and Erdmann [4], which is consistent with current models of postural control based on sensory integration and adaptive motor strategies [15,16].

Post-hoc analyses showed that the posterior body posture pattern differed from both the anterior and neutral patterns in terms of center of pressure sway and stabilogram surface area. In contrast, differences between the anterior and neutral posture patterns were mostly not statistically significant. This indicates that a posterior body alignment may be associated with a different postural control strategy and greater variability of the center of pressure [15,17,18].

Of course, results that are not statistically significant should not be interpreted as conclusive evidence of a lack of association between the variables under study.

From a biomechanical perspective, a posterior body posture pattern may relate to the displacement of the center of pressure relative to the base of support, thereby increasing the demands on the postural control system and resulting in greater center of pressure sway. These mechanisms are consistent with current models of postural stabilization and studies on muscular system adaptation in the correction of pelvic and trunk alignment [15,18].

The relationship of visual conditions did not alter the overall pattern of results, as body posture pattern remained a significant factor under both eyes-open and eyes-closed conditions. At the same time, an increase in center of pressure sway was observed when visual input was removed, which is consistent with the role of sensory integration in postural control [16,17].

In contrast to postural stability parameters, masseter muscle tension did not show statistically significant differences with respect to pelvic type or body posture pattern. This suggests that the activity of the stomatognathic system muscles is not linearly related to global postural alignment under static conditions. This finding is consistent with the results of other authors [19].

The above observations were confirmed by Pearson’s correlation analysis, which did not reveal significant relationships between masseter muscle tension and postural stability parameters. The obtained low correlation coefficients do not support the existence of a linear relationship between these systems under static conditions. However, it should be emphasized that the absence of a linear correlation does not exclude non-linear or condition-dependent relationships [17,20,21].

The lack of observed relationships may result from the multifactorial nature of postural control, which involves the integration of proprioceptive, vestibular, and visual inputs, thereby limiting the relationship of individual muscle groups on global stability parameters [17,20,21]. Additionally, potential interactions between the stomatognathic system and postural control may become apparent primarily under dynamic conditions or during increased functional load.

From a clinical perspective, the results suggest that postural stability in women with pelvic organ prolapse is more closely related to body posture pattern than to pelvic type or masseter muscle activity. Consequently, the assessment and potential correction of global postural organization may represent an important direction for future research [22,23].

Particularly important appears to be the posterior body posture pattern, which was associated with higher stabilometric parameter values. It may reflect a less efficient balance control strategy; however, this interpretation should be limited to static conditions and treated as a hypothesis requiring further investigation [15,17].

The absence of significant differences in masseter muscle activity suggests that the stomatognathic system is not a sensitive indicator of global postural disturbances under static conditions. At the same time, the current literature indicates heterogeneity in findings in this area, which may result from methodological differences and the nature of the tasks being studied [24,25].

### Limitations

Several limitations should be acknowledged. First, the small sample size and uneven subgroup distribution may have reduced statistical power and estimate precision. Therefore, non-significant findings do not necessarily indicate the absence of associations. Second, the cross-sectional design does not allow causal inferences, and potential confounding factors may have influenced the results. Third, the author-developed device used to measure masseter muscle tension requires independent validation and reliability testing. A single measurement per participant further limits confidence in the results. Fourth, the validity and reliability of the force platform measurements and clinical classifications of postural patterns and pelvic type should be established, as measurement and classification errors may have affected the findings. Finally, multiple statistical comparisons may have increased the risk of false-positive results despite the use of Tukey’s post hoc procedure. The analyses should be verified, and appropriate corrections considered.

Overall, these findings are preliminary and require confirmation in larger studies using validated measurement methods, clearly defined primary outcomes, appropriate statistical procedures, and longitudinal designs to establish clinical relevance.

## 5. Conclusions

In this sample of women with pelvic organ prolapse, selected postural stability parameters differed across some postural-pattern categories, while no statistically significant association between pelvic type and postural stability parameters was detected. Weak correlations were observed between selected masseter muscle measurements and postural stability parameters. These findings describe associations within the studied sample and do not establish causal relationships or clinical effectiveness. It should also be noted that the study was conducted on a small group of women, which may have influenced the results obtained and limited the generalizability of the findings to the broader population of women with pelvic organ prolapse.

## Figures and Tables

**Table 1:** Baseline characteristics of the study participants with pelvic organ prolapse. (POP) and the control group (mean ± sd).

Group	Age	Weight	Growth
POP *n* = 31	50 ± 7.07	76.71 ± 11.06	164.42 ± 6.15
control *n* = 30	47 ± 1.41	73.29 ± 11.64	161.1 ± 7.15

**Table 2:** Two-way ANOVA was performed for masseter muscle tension and postural stability parameters under both eyes-open and eyes-closed conditions (pt—pelvis type; bpp—body posture pattern; oeap—open eyes anterior–posterior; oeml—open eyes medio-lateral; oesa—open eyes surface area; ecap—eyes closed anterior–posterior; ecml—eyes closed medio-lateral; ecsa—eyes closed surface area; oeMM—open eyes masseter muscles; ecMM—eyes closed masseter muscles).

oeap						Statistical Power	Presumed Sample S.
	Df	Sum Sq	Mean Sq	F value	Pr(>F)		
pt	2	16,091	8450	0.727	0.4927	0.250	31
bpp	2	153,232	76,616	6.595	0.0048 **		
residuals	26	302,044	11,617				
oeml						Statistical Power	Presumed Sample S.
	Df	Sum Sq	Mean Sq	F value	Pr(>F)		
pt	2	5462	2731	1.153	0.3314	0.192	31
bpp	2	20,359	10,180	4.296	0.0244 *		
residuals	26	61,608	2370				
oesa						Statistical Power	Presumed Sample S.
	Df	Sum Sq	Mean Sq	F value	Pr(>F)		
pt	2	165,425	82,712	0.562	0.5768	0.166	31
bpp	2	1,510,425	755,213	5.133	0.0132 *		
residuals	26	3,825,625	147,139				
ecap						Statistical Power	Presume Sample S.
	Df	Sum Sq	Mean Sq	F value	Pr(>F)		
pt	2	52,316	26,158	0.638	0.536	0.194	31
bpp	2	399,476	199,738	4.872	0.016 *		
residuals	26	1,066,029	41,001				
ecml						Statistical Power	Presumed Sample S.
	Df	Sum Sq	Mean Sq	F value	Pr(>F)		
pt	2	3550	1775	0.487	0.6200		
bpp	2	76,659	38,330	10.515	0.0004 ***	0.367	31
residuals	26	94,776	3645				
ecsa						Statistical Power	Presumed Sample S.
	Df	Sum Sq	Mean Sq	F value	Pr(>F)		
pt	2	609,387	304,694	1.151	0.3318		
bpp	2	3,606,006	1,803,003	6.813	0.0041 **	3.985	31
residuals	26	6,881,002	264,654				
oeMM						Statistical Power	Presumed Sample S.
	Df	Sum Sq	Mean Sq	F value	Pr(>F)		
pt	2	14.42	7.208	1.569	0.227		
bpp	2	12.33	6.167	1.342	0.279	0.119	31
residuals	26	119.44	4.594				
ecMM						Statistical Power	Presumed Sample S.
	Df	Sum Sq	Mean Sq	F value	Pr(>F)		
pt	2	15.34	7.670	0.669	0.521		
bpp	2	8.38	4.189	0.365	0.697	0.073	31
residuals	26	298.10	11.465				

Signif. codes: 0 ‘***’ 0.001 ‘**’ 0.01 ‘*’

**Table 3:** The eta-squared values and 95% CIs are as follows.

	pt	bpp
	eta_squared	eta_squared
oeap	0.05 [0.00, 0.25]	0.34 [0.05, 0.55]
oeml	0.08 [0.00, 0.29]	0.25 [0.00, 0.48]
oesa	0.04 [0.00, 0.22]	0.28 [0.02, 0.51]
ecap	0.05 [0.00, 0.23]	0.27 [0.01, 0.50]
ecml	0.04 [0.00, 0.21]	0.45 [0.14, 0.64]
ecsa	0.08 [0.00, 0.29]	0.34 [0.05, 0.56]
oeMM	0.11 [0.00, 0.33]	0.09 [0.00, 0.31]
ecMM	0.05 [0.00, 0.24]	0.03 [0.00, 0.19]

**Table 4:** Tukey’s post-hoc test was performed for body posture pattern and masseter muscles tension, as well as postural stability parameters under both eyes-open and eyes-closed conditions (bpp—body posture pattern; oeap—open eyes anterior–posterior; oeml—open eyes medio-lateral; oesa—open eyes surface area; ecap—eyes closed anterior–posterior; ecml—eyes closed medio-lateral; ecsa—eyes closed surface area; open eyes masseter muscles; oeMM—open eyes masseter muscle; ecMM—eyes closed masseter muscles).

oeap-bpp	*p*-Value
neutral-anterior	0.9591
posterior-anterior	0.0053
posterior-neutral	0.0080
**oeml-bbp**	
neutral-anterior	0.7658
posterior-anterior	0.0215
posterior-neutral	0.0539
**oes-bbp**	
neutral-anterior	0.8931
posterior-anterior	0.0127
posterior-neutral	0.0238
**ecap-bbp**	
neutral-anterior	0.9993
posterior-anterior	0.0198
posterior-neutral	0.0208
**ecml-bbp**	
neutral-anterior	0.8622
posterior-anterior	0.0004
posterior-neutral	0.0010
**ecsa-bbp**	
neutral-anterior	0.9167
posterior-anterior	0.0043
posterior-neutral	0.0077
**ocMM-bbp**	
neutral-anterior	0.5698
posterior-anterior	0.7460
posterior-neutral	0.3860
**ecMM-bbp**	
neutral-anterior	0.9989
posterior-antrior	0.7220
posterior-neutral	0.7066

Signif. codes: 0 ‘***’ 0.001 ‘**’ 0.01 ‘*’

**Table 5:** Descriptive statistics were calculated for masseter muscle tension and postural stability parameters under both eyes-open and eyes-closed conditions (bbp—body posture pattern; oeap—open eyes anterior–posterior; oeml—open eyes medio-lateral; oesa—open eyes surface area; ecap—eyes closed anterior–posterior; ecml—eyes closed medio-lateral; ecsa—eyes closed surface area; oeMM—open eyes masseter muscles; ecMM—eyes closed masseter muscles).

bbp-oeap	Mean	Std	Min	Max	Q25	Q50	Q75
neutral	198.6429	59.9135	101	290	147	199.50	238.75
anterior	208.1429	80.9195	118	362	137.75	194	228.75
posterior	442.0000	304.5373	124	731	297.50	471	601
bbp-oeml							
neutral	135.5714	48.6189	84	260	106	119.5	166.25
anterior	134.2857	32.9648	93	209	109.50	125	151.25
posterior	220.6667	106.4017	120	332	165	210	271
bbp-oesa							
neutral	377.2857	281.4590	115	1163	167.75	316.50	474.25
anterior	376	204.5607	133	792	224.50	304.50	520
posterior	1134.3333	1089.0401	171	2316	543.50	916	1616
bbp-ecap							
neutral	283.2857	149.9823	127	597	178.50	218.50	338
anterior	307.1429	203.4071	121	949	206.25	246	315.5
posterior	699.6667	346.6155	300	918	590.50	881	899.5
bbp-ecml							
neutral	146.2143	45.2585	93	232	106.50	137.50	174
anterior	141.8571	39.6190	99	227	108.25	136.50	157.75
posterior	315	155.9134	135	408	268.50	402	405
bbp-ecsa							
neutral	514.6429	425.6643	140	1325	214.25	318	803.5
anterior	505.2857	327.9199	173	1127	252.75	391.5	642
posterior	1742	1270.6986	387	2907	1159.50	1932	2419.5
bbp-oeMM							
neutral	4.25	2.57674	1.55	9.5	2.15	3.825	5.15
anterior	3.0214	1.7327	0.00	6.9	1.8875	2.7	3.65
posterior	2.4	1.7521	0.60	4.1	1.55	2.5	3.3
bbp-ecMM							
neutral	4.0071	3.4135	1	11.9	1.9375	2.5	4.2875
anterior	3.6164	3.4550	0	12.6	1.4125	2.55	4.1
posterior	2.383333	2.0678	0	3.7	1.7250	3.45	3.575

## Data Availability

The data presented in this study are available on request from the corresponding author due to privacy.
